# Retroperitoneal Necrotizing Fasciitis Following Prolonged Physical Activity: A Case Report

**DOI:** 10.5811/cpcem.34862

**Published:** 2025-04-01

**Authors:** Jordan R. Pollock, Edmundo Chantler, Bhavesh Patel, Nelly Tan, Wayne Martini

**Affiliations:** * Mayo Clinic Arizona, Department of Radiology, Phoenix, Arizona; † Mayo Clinic Arizona, Department of Emergency Medicine, Phoenix, Arizona; ‡ Mayo Clinic Arizona, Department of Critical Care Medicine, Phoenix, Arizona

**Keywords:** acute kidney injury, retroperitoneal necrotizing fasciitis, necrotizing fasciitis, pyomyositis, rhabdomyolysis

## Abstract

**Introduction:**

Retroperitoneal necrotizing fasciitis is a rare, rapidly progressive, and often fatal infection of the retroperitoneum. In many cases the source of infection is unclear, and cutaneous signs of necrotizing fasciitis may be absent.

**Case Report:**

We present the case of a 64-year-old female with a history of hypertension, hyperlipidemia, and breast cancer who developed acute kidney injury (AKI) and retroperitoneal necrotizing fasciitis following a 20-mile bike ride. The patient’s initial symptoms included severe muscle aches, nausea, vomiting, and flank pain. Diagnostic imaging and laboratory results indicated myositis and severe AKI. Despite aggressive treatment with antibiotics, intravenous fluids, and pain management, the patient developed septic shock and multiorgan failure, ultimately leading to her death.

**Conclusion:**

This case highlights the rapid progression and complexity of managing necrotizing fasciitis and AKI in the context of rhabdomyolysis. Early recognition and aggressive management are crucial in cases of suspected necrotizing fasciitis and AKI. Patients may not initially present with cutaneous findings suggestive of necrotizing fasciitis. Early involvement of a multidisciplinary team can improve patient outcomes in complex and rapidly deteriorating patients.

## INTRODUCTION

Retroperitoneal necrotizing fasciitis is a rare, rapidly progressive and often fatal infection of the retroperitoneum. This infection has a high mortality rate, estimated to be 40–60%.^1^ In many cases the source of infection is unclear. A systematic review of peritonitis caused by streptococcus found that 69% of patients developed the infection from an unknown source, 16% of patients’ source of infection was from ascending vaginal infection, 9% from a droplet infection, and 6% from pharyngitis.^2^

Cutaneous manifestations of necrotizing fasciitis, such as necrosis and erythema, will often present in patients with a defined infectious source such as a wound or surgical site. However, according to another study, more than half of patients with necrotizing fasciitis had no defined portal of entry and arrived to the emergency department (ED) with the main symptom of increasingly severe pain. This pain can begin at a site of recent trauma such as a joint injury, hematoma, or muscle strain.^3^

## CASE REPORT

A 64-year-old female with a history of hypertension managed with lisinopril, hyperlipidemia, and breast cancer status post mastectomy and current letrozole use presented to the ED with severe muscle aches, nausea, vomiting, diarrhea, decreased urine output, frontal headache, and flank pain. She was physically active most days of the week including walking, running, and occasional bicycle riding. Four days prior to arrival, the patient had completed a challenging 20-mile bike ride on a summer day. Three days prior to arrival, she developed severe muscle aches. One day before arrival, she experienced diarrhea, nausea, vomiting, and headache. The patient came to the ED due to these symptoms, in addition to left flank pain and decreased urine output. (See Figure for a timeline of patient illness.)

The patient had never smoked or used smokeless tobacco. She consumed about 10 alcoholic drinks per week. She had a family history of hypertension. She had recently traveled from Utah to Arizona after her biking trip. On presentation, she appeared anxious with a heart rate of 70 beats per minute and respirations of 20 breaths per minute; she was afebrile at 36.6 °Celsius and hypertensive to 138/99 millimeters of mercury. Her oxygen saturation was 100% without supplemental oxygen use. The patient rated her flank pain a 10/10. On physical examination, the patient had a normal cardiovascular and pulmonary exam. There was moderate pain to palpation of the left middle and left lower flank, with mild right lower quadrant abdominal tenderness.

Initial laboratory results indicated a white blood cell count (WBC) of 6.1 × 10^9^ cells per liter (L) (reference range: 4.5–11.0 × 10^9^/L) and hemoglobin of 13.4 grams per deciliter (g/dL) (11.6–15.0 g/dL). She had a sodium of 129 millimoles (mmol) per L (135–145 mmol/L), chloride 92 mmol/L (98–107 mmol/L), bicarbonate 18 mmol/L (22–29 mmol/L), and anion gap of 19 (7–15). She had a severe acute kidney injury (AKI) with a creatinine of 4.25 milligrams (mg)/dL) (patient baseline 0.8, reference range 0.59–1.04 mg/dL) and an elevated blood urea nitrogen of 45 mg/dL (6–21 mg/dL). Her lactate was elevated to 4.1 mmol/L (0.5–2.2 mmol/L) and creatine kinase (CK) of 200 units (U)/L (2–192 U/L). Influenza A, influenza B, respiratory syncytial virus, and coronavirus polymerase chain reaction tests were negative.

**Image 1:**
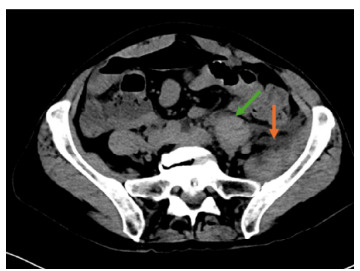
Lower sections of computed tomography abdomen and pelvis demonstrates heterogenous hyper-attenuation of the left psoas major muscle (green arrow) and iliacus muscle (orange arrow).

Diagnostic imaging with computed tomography of the abdomen and pelvis without contrast due to her kidney injury revealed heterogeneous hyperattenuation and asymmetric enlargement of the left quadratus lumborum muscle, external oblique muscle, left psoas major muscle, and iliacus muscle (Image 1).

**CPC-EM Capsule**

What do we already know about this clinical entity?

*Retroperitoneal necrotizing fasciitis is a rapidly progressive disease associated with a high morbidity and mortality*.

What makes this presentation of disease reportable?

*This is a rare case of retroperitoneal necrotizing fasciitis without a clear infectious source and an initially non-specific disease presentation*.

What is the major learning point?

*Retroperitoneal necrotizing fasciitis does not always have a clear infectious source and should*

*be considered when in the setting of severe pain or organ failure*.

How might this improve emergency medicine practice?

*Early recognition of retroperitoneal necrotizing fasciitis requires a broad differential and recognition of rapidly progressive multisystem organ failure*.

The patient was admitted to internal medicine for treatment of her AKI, elevated lactic acid, and pain, and she was treated with aggressive antibiotics including cefepime 1 gm intravenously (IV), doxycyline 100 mg IV, linezolid 600 mg IV, and metronidazole 500 mg IV. She was also treated with IV fluid resuscitation and pain management with morphine 2 mg and multiple 1 mg doses of hydromorphone. She was also given hydrocortisone 50 mg and IV immune globulin (human) 10% infusion 20 g. The patient started continuous renal replacement therapy due to her AKI and subsequent severe metabolic acidosis.

**Image 2:**
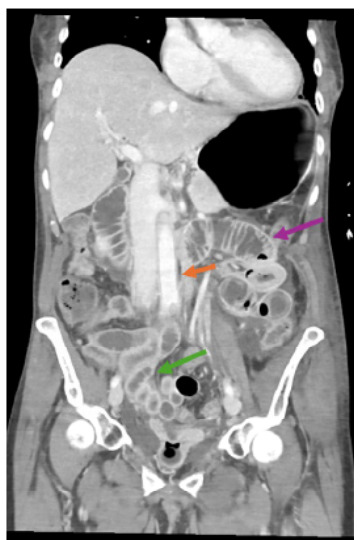
Post contrast computed tomography abdomen and pelvis performed one day after the initial presentation demonstrated the muscular findings; in addition, there was new diffuse thickening of the aorta concerning for aortitis (orange arrow), new proximal small bowel dilatation with areas of thickening (purple arrow) and the distal small bowel hyper-enhancing wall (green arrow) concerning for sequelae of active septic shock.

Five hours after admission, the patient developed rapidly progressive multiorgan failure that presented as a possible stroke, after which she was intubated, and additional imaging was obtained (Image 2). Her CK increased from 200 to 5,535 U/L, serum creatinine from 4.25 to 4.41 mg/dL, blood urea nitrogen from 45 to 51 mmol/L, and WBC decreased from 6.9 to 3.7 × 10^9^ cells/L.

Surgical consultation was obtained due to suspicion of necrotizing fasciitis, for which the patient was deemed inoperable due to the extensive organ involvement of the infection. A muscle biopsy was obtained and confirmed to be group A *Steptococcus pyogenes*. The patient was transitioned to comfort care after family discussions of these findings. She died shortly thereafter due to multiorgan failure and septic shock.

## DISCUSSION

In patients with no defined infectious source, the severe pain associated with necrotizing fasciitis can precede cutaneous evidence of infection by 12–24 hours. In our case, the patient presented with increasingly severe left flank pain followed by cutaneous findings of necrotizing fasciitis after admission to the hospital. She did not present with an increased WBC on initial presentation, possibly due to her use of letrozole for breast cancer.

The treatment of necrotizing fasciitis includes early recognition, administration of broad spectrum IV antibiotics, and surgical debridement.^4^ Retroperitoneal necrotizing fasciitis is a rare, aggressive infection associated with a high mortality. A case report of a young, healthy, 33-year-old female patient is similar to our case. She presented to the ED with generalized abdominal pain suspected to be due to gastroenteritis, and a CT demonstrated intrabdominal fluid likely secondary to a ruptured corpus luteal cyst. The patient returned with worsening pain, with repeat CT demonstrating worsening free fluid and evidence of peritonitis. Subsequently laparoscopy confirmed peritonitis with no identified infectious source, with cultures positive for *group A S. pyogenes*.^5^ Intra-abdominal necrotizing fasciitis should be part of a broad differential considered in the ED to reduce the mortality and morbidity associated with this condition. One tool is the Laboratory Risk Indicator for Necrotizing Fasciitis (LRINEC) score to distinguish necrotizing fasciitis from severe cellulitis or abscess, which takes into account C-reactive protein, WBC, hemoglobin, sodium, creatinine, and glucose.^6^

In another case, a 33-year-old man had a three-week history of back ache due to trauma, followed by fever, vomiting, and severe left flank pain, which was then diagnosed as retroperitoneal necrotizing fasciitis due to *Escherichia coli*. The source for this patient was unclear, similar to our patient.^7^ However, our patient had gone mountain biking for 20 miles, which could have been a source of trauma and back pain for her. In cases of trauma and hematoma formation, necrotizing fasciitis has been known to infect these areas. For example, a 26-year-old man presented with severe pain in his right biceps, which he attributed to a muscle tear, and was diagnosed as a muscle belly tear with hematoma formation. The patient returned four days later with necrotic skin and erythema of the upper extremity and grew group F β-hemolytic streptococci and *Bacteroides*.^8^

In our case the source of the patient’s retroperitoneal necrotizing fasciitis remains unclear, but contributing factors included her recent strenuous bike ride and use of letrozole for breast cancer. Kejela et al described 14 cases of retroperitoneal necrotizing fasciitis, with infection sources ranging from Fournier gangrene, fistula, and abscess to perforated diverticulitis, renal stone, and “none.”^9^

## CONCLUSION

This case highlights the rapid progression and complexity of managing necrotizing fasciitis and acute kidney injury in the context of rhabdomyolysis. Early recognition and aggressive management are crucial in cases of suspected necrotizing fasciitis and AKI. Early involvement of a multidisciplinary team can improve patient outcomes in complex and rapidly deteriorating patients.

## Figures and Tables

**Figure:**
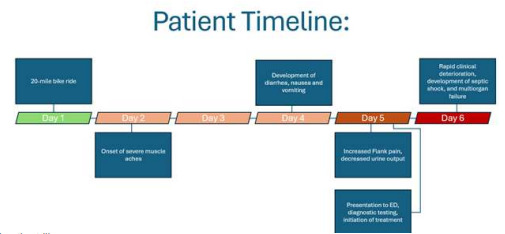
Timeline of patient illness. *ED*, emergency department.
